# Quality of Life and General Health in Pregnant Women Conceived with Assisted Reproduction Technologies: A Case-Control Study

**DOI:** 10.22074/ijfs.2020.5684

**Published:** 2019-11-11

**Authors:** Mohammad Sarafraz Yazdi, Roya Nasiri, Masoud Gharaei Jomei, Saman Sarafraz Yazdi

**Affiliations:** 1 Department of Internal Medicine, Islamic Azad University, Mashhad Branch, Mashhad, Iran; 2 Department of Obstetrics and Gynecology, Islamic Azad University, Mashhad Branch, Mashhad, Iran

**Keywords:** Assisted Reproduction Technology, General Health, Infertility, Quality of Life

## Abstract

**Background:**

Infertility affects different aspects of life including the quality of life (QOL) in infertile couples. Many infertile couples conceive via using assisted reproduction technologies (ART). However, the effect of preg- nancy and childbearing on QOL is not known in these couples. This study aimed to evaluate QOL and general health during pregnancy and after successful treatment of infertility, in women conceived with ART.

**Materials and Methods:**

In this case-control study, QOL and general heath were evaluated in 40 women conceived with ART and 40 women who conceived spontaneously and served as the control group. WHO quality of life- BREF (WHOQOL-BREF) inventory was used to evaluate QOL and General Health Questionnaire-28 (GHQ-28) was applied to evaluate general health. These two questionnaires were completed in the first and second trimester of pregnancy and results were compared between the two groups.

**Results:**

Mean age of women was 29.4 ± 4.4 and 29.6 ± 5 years in ART and control group, respectively. QOL in women conceived with ART was similar to QOL in the control group in the first and second trimester of pregnancy while general health score (distress level) in women conceived with ART was significantly higher than that of the control group in both trimesters. Although distress level decreased in the second trimester in ART group, but yet, it was higher than that recorded for the control group.

**Conclusion:**

After pregnancy, QOL in women conceived with ART is similar to women conceived spontaneously. However, these women experience higher distress level in the first and second trimester of pregnancy compared to women conceived spontaneously.

## Introduction

Infertility is defined by failure of getting pregnant after at least one year of regular and unprotected intercourse (1). Worldwide, about 1 out of 4 couples in developing countries, 1 out of 8 in developed countries and globally about 8-12% of couples are affected by primary or secondary infertility (2). In about 40% of infertile couples male factors, in 40% of them female factors and in 20% of cases combinations of both or unknown causes, are responsible for infertility (3). 

Prevalence of primary infertility in a population-based study in an urban population in Iran was 17.3% which is higher than global infertility rate. In Iranian couples, female (56.1%) and male factors (29.1%) were the most common causes of infertility, followed by unexplained infertility (14.4%). Among female factors of infertility, ovulation disorders (39.7%) were the most common cause (4). 

Infertility can profoundly affect different aspects of life in infertile couples and due to its social, cultural and economic problems, it produces a sever crisis in infertile couples’ life and causes severe distress and psychological (anxiety, depression, etc.) (5, 6) and financial problems (7). Infertility may also cause problems in couples relationship or even lead to divorce (7, 8). Infertility stigma for women in regions with traditional cultures like Middle East countries, is more prominent and stressful and causes various problems for them (9).

Above-mentioned factors affect deeply the quality of life (QOL) and general health in infertile couples (6, 10). Several studies investigated the relationship between depression, anxiety, QOL, general health and marital satisfaction in infertile couples and socio-demographic determinants of QOL (11-14).

Most of studies showed impaired QOL and general health in infertile couples where QOL was affected by factors such as the duration of infertility, age, education, income, residential place and cause of infertility (6, 10, 15-17). A previous study showed that more than half of infertile women have a degree of general health disorder (6). In a study, Maroufizadeh et al. (11) found that QOL in infertile couples was influenced by their own and their spouses’ depression. In another study, Maroufizadeh et al. (13) showed that marital satisfaction in infertile patients was affected by their own and their spouses’ perceived stress.

Following successful progresses in infertility treatment and achieving pregnancy, it is assumed that the abovementioned problems may decrease and QOL may improve (18). Nevertheless, following successful conception, due to high risk pregnancy and concern of continuity of pregnancy distress may increase resulting in decrement of QOL. However, limited studies evaluated QOL and general health of infertile couples after conception and during pregnancy.

The main goal of this study was to evaluate QOL and general health in pregnant women conceived with assisted reproductive technology (ART).

## Materials and Methods

This case-control study was conducted during 2013- 2014 in a private clinic in Mashhad, Iran and 40 pregnant women conceived with ART and 40 pregnant women who conceived spontaneously were included. 

Pregnant women who conceived with one of the ART methods including *in vitro* fertilization (IVF) or intra cytoplasmic sperm injection (ICSI), those who did not have a previous history of successful pregnancy or children and were in the first trimester (gestational age under 12 weeks) of pregnancy, were included in case group. Pregnant women, who did not have previous successful pregnancy or children (nulliparous) but conceived spontaneously and were in the first trimester of pregnancy, were enrolled as control group. The two groups were matched for age, education, income and gestational age.

Pregnant women with a history of chronic diseases, diabetes, cardiovascular diseases, seizure, or addiction and those with a history of psychiatric disorders as well as those who did not sign the informed consent, were excluded from the study.

To evaluate QOL, a Persian version of WHO quality of life- BREF questionnaire (WHOQOL-BREF) was used. Also, a Persian version of General Health Questionnaire-28 (GHQ-28) was used to evaluate general health in the participants. In the present study, participants completed these two questionnaires twice (once in the first and once in the second trimester of pregnancy).

WHOQOL-BREF inventory has 26 items and four domains including physical health, psychological health, social relationship and environment. Reliability and validity of the Persian version of WHOQOL-BREF was previously evaluated and approved (19). Two scoring systems are used in WHOQOL-BREF. In the first method, the inventory is scored between 0 and 100 and in the second method, it is scored between 4 and 20. In the current study, both methods were used but in the Tables, only results from the first method are presented. 

GHQ-28 is a self-administered inventory with 28 items that has been developed for screening of emotional distress and possible psychiatric morbidity. GHQ-28 evaluates psychological well-being in four subscales namely, somatic symptoms, anxiety/insomnia, social dysfunction and severe depression. Each subscale has seven questions and each item has four optional responses scored 0 to 3 as follows; score 0: “not at all” score 1: “no more than usual”; score 2: “rather more than usual” and score 3: “much more than usual.” The total score of the GHQ-28 ranges from 0 to 84 and a higher score indicates a higher distress level. In each subscale, a score >6 was considered “abnormal condition”.

Validity and reliability of the Persian version of GHQ28 was previously assessed and confirmed (20). Two inventories were completed by participants in the first and second trimester of pregnancy. Demographic information including age, gestational age, education and economic status, etc. was recorded in a separate form.

### Data analysis

Considering Nilforooshan et al. (21) study that mean of QOL score in case and control group was 170.52 ± 18.17 and 182.22 ± 18.08, and by considering 95% CI and power of 80%, sample size was calculated by following formula:

$$n=\frac{(S_{1}^{2}+S_{2}^{2}){\left(Z_{1-\frac{\alpha }{2}}+Z_{1-\beta }\right)}^{2}}{{\left({\overset{-}{X}}_{1}-{\overset{-}{X}}_{2}\right)}^{2}}=\frac{(18.17^{2}+18.08^{2}){\left(1.96+0.84\right)}^{2}}{{\left(182.22-170.52\right)}^{2}}=37/62$$

Sample size of 40 was considered in each group. Obtained data was analyzed using SPSS software version 22.00 for Windows (Armonk, NY: IBM Corp.) and STATISTICA Ver. 10.00. Numerical data are presented as mean ± standard deviation and categorical data as numbers/percentages. Normality of data was assessed by Kolmogorov-Smirnov test with the correction of Lilliefors. 

For comparison of data with normal distribution between the two groups, Student’s t test was used and for comparison of data obtained in the first and second trimester of pregnancy, paired t test was used. For comparison of data without normal distribution, nonparametric Mann-Whitney U test was applied. To compare qualitative and categorical data such as education and job between the two groups, Chi-square test was applied. A P≤0.05 was considered significant.

### Ethical considerations

Institutional Review Board at Mashhad Branch of Islamic Azad University approved the study protocol and all participants signed written informed consent before enrollment (IR.IAU.NEYSHABUR.REC.1398.008).

### Results

Eighty women in the first trimester of pregnancy participated in this study; of them, 40 conceived with ART and 40 conceived spontaneously (control group). Mean age of all women was 29.5 ± 4.7 years. Demographic characteristics of pregnant women in two study groups are depicted in Table 1. There were no significant differences in age, gestational age, education, job and income between the two groups.

There were no significant differences in QOL score between ART and control group neither in the first trimester nor in the second trimester of pregnancy. Also, there were no significant differences between the two groups in none of the four QOL sub-domains in the first and second trimesters of pregnancy. In the second trimester of pregnancy, QOL improved significantly in both groups compared to the first trimester (P=0.006 and P=0.03, respectively) while the differences in the four subdomains of QOL were not significantly different between the first and second trimesters, in each group (Table 2).

There was a significant difference in general health between the women conceived with ART and the control group in a way that general health score of women conceived with ART was significantly higher than those conceived spontaneously in the first as well as the second trimester of pregnancy (P<0.001). Also, a significant difference was observed between the two groups in all subscales of GHQ-28 in the first trimester of pregnancy while in the second trimester a significant difference was only found in somatic symptoms and anxiety (P=0.001 and 0.009, respectively, Table 3)

In the second trimester of pregnancy, in the ART group, general health score and all its subscales except for somatic symptoms, were significantly lower than those of the first trimester of pregnancy while in the control group, total general health score and all its subscales were significantly higher than those of the first trimester of pregnancy (Table 3).

**Table 1:** Demographic characteristics of study participants in two study groups


Parameter		ART pregnancy group	Spontaneous pregnancy group	P value

Age (Y)		29.42 ± 4.39	29.57 ± 5.02	0.47
Education				
	Primary	13 (32.5)	10 (25)	
	High school diploma	11 (27.5)	13 (32.5)	
	University education	16 (40)	17 (42.5)	0.74
Job				
	Housewife	30 (75)	29 (72.5)	
	Employed	10 (25)	11 (27.5)	0.79
Income^*^				
	Below 5000	6 (15)	7 (17.5)	
	5000-10000	14 (35)	16 (40)	
		10000-15000	12 (30)	12 (30)	
	More than 15000	8 (20)	5 (12.5)	0.82
Gestational age (weeks)		9.87 ± 1.91	9.55 ± 2.11	0.55


Data are presented as mean ± SD or n (%). *; Thousands rials and ART; Assisted reproductive technology.

**Table 2:** Quality of life score and scores of its four domains in two study groups in the first and second trimesters of pregnancy (0-100)


WHOQOL-BREF domain		ART pregnancy group	Spontaneous pregnancy group	P value	95% CI

First trimester of pregnancy					
	Physical health	50.71 ± 12.05	51.61 ± 11.91	0.74	-4.44, 6.22
	Psychological health	57.08 ± 19.29	60.83 ± 19.28	0.38	-4.83, 12.33
	Social relationship	62.08 ± 15.20	65.83 ± 14.71	0.26	-2.91, 10.41
	Environment	60.31 ± 12.40	63.35 ± 14.20	0.31	-2.88, 8.98
	Overall feeling	66.25 ± 25.50	68.43 ± 23.34	0.69	-8.69, 13.07
Second trimester of pregnancy					
	Physical health	51.42 ± 11.17	52.58 ± 12.68	0.66	-4.16, 6.48
	Psychological health	59.37 ± 18.62	60.20 ± 20.71	0.85	-7.93, 9.60
	Social relationship	61.66 ± 13.96	64.79 ± 13.80	0.31	-3.05, 9.30
	Environment	60.78 ± 14.65	62.50 ± 14.41	0.59	-4.75, 8.19
	Overall feeling	73.43 ± 22.32^*^	74.06 ± 19.69^*^	0.89	-8.74, 9.99


Data are presented as mean ± SD or n (%). *; Thousands rials and ART; Assisted reproductive technology.

**Table 3:** GHQ-28 and its subscales scores in two study groups in the first and second trimesters of pregnancy


GHQ-28 domain		ART pregnancy groupn=40	Spontaneous pregnancy groupn=40	P value	95% CI

The first trimester of pregnancy					
	Somatic symptoms	8.70 ± 3.00	5.60 ± 3.30	0.001^*^	-4.50, -1.69
	Anxiety and insomnia	9.15 ± 3.36	5.80 ± 2.52	0.001^*^	-4.67, -2.02
	Social dysfunction	8.55 ± 3.28	6.22 ± 2.61	0.001^*^	-3.64, -1.00
	Severe depression	6.85 ± 3.14	5.47 ± 2.83	0.04^*^	-2.70, -0.04
	Total general health	33.25 ± 7.41	23.10 ± 5.68	0.001^*^	-13.09, -7.20
The second trimester of pregnancy					
	Somatic symptoms	9.65 ± 3.18^†^	6.80 ± 3.39^†^	0.001^*^	-4.31, -1.38
	Anxiety and insomnia	7.80 ± 2.20^†^	6.35 ± 2.64^†^	0.009^*^	-2.53, -0.36
	Social dysfunction	7.52 ± 2.63^†^	6.60 ± 2.76^†^	0.12	-2.12, 0.27
	Severe depression	6.05 ± 2.36^†^	6.05 ± 3.39^†^	0.99	-1.30, 1.30
	Total general health	31.02 ± 5.54^†^	25.80 ± 6.18^†^	0.001^*^	-7.83, -2.61


Data are presented as mean ± SD. ART; Assisted reproductive technology, CI; Confidence interval, *; Significant difference, GHQ-28; General Health Questionnaire-28, and †; Significant changes compared to the first trimester of pregnancy in the same group.

**Table 4:** Prevalence of psychiatric disorders based on GHQ-28 results in two study groups in the first and second trimesters of pregnancy


GHQ-28 domain		ART pregnancy groupn=40	Spontaneous pregnancy groupn=40	P value

The first trimester of pregnancy				
	Somatic symptoms disorder	32 (80)	12 (30)	<0.001^*^
	Anxiety and insomnia disorder	30 (75)	14 (35)	<0.001^*^
	Social function disorder	29 (72.5)	20 (50)	0.03^*^
	Severe depression disorder	22 (55)	9 (22.5)	<0.001^*^
	Total general health disorder	38 (95)	21 (52.5)	<0.001^*^
The second trimester of pregnancy				
	Somatic symptoms disorder	33 (82.5)	18 (45)	<0.001^*^
	Anxiety and insomnia disorder	27 (67.5)	17 (42.5)	0.02^*^
	Social function disorder	26 (65)	19 (47.5)	0.11
	Severe depression disorder	18 (45)	12 (30)	0.16
	Total general health disorder	39 (97.5)	26 (65)	<0.001^*^


Data are presented as n (%). *; Significant difference, ART; Assisted reproductive technology, and GHQ-28; General Health Questionnaire-28

Somatic symptoms score in the ART group was significantly higher in the second trimester of pregnancy (P<0.001). However, the difference in GHQ-28 between the two study groups was significant and distress in the ART group was higher compared to the control group, in the second trimester of pregnancy (Table 4). Prevalence of general health disorders based on GHQ-28 in two study groups has been shown in Table 4.

## Discussion

This study found that QOL in pregnant women conceived with ART was similar to women conceived spontaneously, in the first and second trimesters of pregnancy while general health in ART group was significantly superior to control group in the first and second trimesters. Women conceived with ART had significantly higher somatic symptoms, anxiety, social dysfunction and depression compared to the control group, in the first trimester of pregnancy. In the second trimester of pregnancy, all GHQ-28 subscales were significantly reduced compared to the first trimester in the ART group while at the same time, distress increased in the control group. In the first trimester of pregnancy, in women conceived with ART, stress, anxiety and depression increase probably due to uncertainty about the continuity of pregnancy and in the second trimester, this uncertainty about stability of pregnancy decreases which may lead to reduced distress and anxiety. 

Due to infertility and probably repeated treatment failures, couples face different problems such as financial problems and difficulties in social relations that affect different aspects of their life. Infertility has negative psychological effects such as anxiety, depression (5, 11) stress, hopelessness, etc. which reduce QOL of infertile couples. 

Maroufizadeh et al. (14) showed that both men’s and women’s anxiety affect the marital satisfaction. Also, they found that in infertile couples, women’s anxiety has a significant effect on their partner marital satisfaction. They showed that in infertile couples, marital satisfaction of each member of each couple has an effect on his/her own depression. They also found that men’s marital satisfaction has a significant effect on their partner depression symptoms while the wives’ marital satisfaction has no effect on husbands’ depressive symptoms (12).

Previous studies showed poor QOL in infertile couples in Iran and other countries in the world (15, 21-26). These studies showed that depression, anxiety, failure of previous treatments, female gender, lower educational level, younger age and unknown cause of infertility were associated with lower QOL (21, 27) while higher educational level, social support and coping strategy increased QOL in these couples (15). However, duration of infertility has no effect of QOL (27). 

However, successful infertility treatment and conception may restore reduced QOL and increased distress level during pregnancy. In a study conducted in Canada, QOL was evaluated in 243 women conceived with ART and 3,309 women with spontaneous conception before the 25^th^ week of pregnancy and during the 34^th^-36^th^ weeks of gestational age as well as four months postpartum by using SF-12 questionnaire. This study reported lower physical and mental health for women conceived with ART during pregnancy (before the 25^th^ week and during the 34^th^-36<sup>th</sup> weeks of pregnancy) compared to women conceived spontaneously while these indices were equal between the two groups four months postpartum (28). Findings of that study are different from ours as we did not observe any difference between the two groups in QOL in the first and second trimester. The reason of such discrepancy may be application of different questionnaires for evaluation of QOL (SF-12 vs. WHOQOL-BREF). Also, postpartum QOL was not evaluated in the present study.

Gameiro et al. (29) study done in Portugal, evaluated QOL in 66 women conceived with ART and compared it with QOL determined for 70 women conceived spontaneously, during the 24^th^ week of pregnancy and four months postpartum by using WHOQOL-BREF inventory. In their study, physical health in women of both groups was similar during pregnancy and improved four months postpartum in both groups, although its improvement in the ART group was better. Psychological health score in women of the ART group during pregnancy was higher than the control group and four months postpartum reduced more than the control group as well. The same changes were observed for psychological health in men of the ART group. Although similar to our study, Gameiro et al. (29) used WHOQOL-BREF inventory but the findings were to some extent different that may be due to the larger sample size of their study.

Ahmadi et al. (30) evaluated QOL in 86 women conceived with ART and 162 women with natural conception by using SF-36 inventory in the last trimester of pregnancy and one month postpartum. In this study, subdomains of physical functioning, role physical, general health and social functioning were significantly different between the two groups before childbirth and improved one month postpartum in both groups except for social functioning that did not improve in control group significantly. However, improvements in all QOL measures in the ARTs group, were greater, expect for general health, than the control group. Ahmadi et al. (30) also applied SF-36 inventory which is different from what we used in the present work.

A study in Slovenia showed that women conceived with ART had positive emotion that improved by progression of pregnancy despite the existence of more medical problems during pregnancy. However, they tend to social isolation (31). They used QOL scale to evaluate QOL which has different items compared to WHOQOL-BREF inventory that was used in our study.

The main limitation of the current study was the small sample size and lack of assessment of QOL and general health in the last trimester of pregnancy and postpartum. Cross- sectional design of the study, use of self-report questionnaire and lack of evaluation of psychological factors such as depression, anxiety, stress and self-esteem, were other limitations of the current study.

Future studies with larger sample size which assess QOL and general health using other valid, approved inventories in all trimesters of pregnancy and postpartum are suggested to be conducted to identify possible changes in QOL in the third trimester of pregnancy and during postpartum.

## Conclusion

It seems that in infertile women following treatment and after successful conception and during pregnancy, QOL is similar to women conceived spontaneously and is not different. Although during pregnancy these women have high distress levels but by progression of pregnancy and increasing certainty about pregnancy, distress level reduces.
